# Metastatic “Burned Out” Seminoma Causing Neurological Paraneoplastic Syndrome—Not Quite “Burned Out”

**DOI:** 10.3389/fneur.2018.00020

**Published:** 2018-01-30

**Authors:** Yuval Freifeld, Payal Kapur, Ritika Chitkara, Francesca Lee, Pravin Khemani, Aditya Bagrodia

**Affiliations:** ^1^Department of Urology, University of Texas Southwestern Medical Center at Dallas, Dallas, TX, United States; ^2^Department of Pathology, University of Texas Southwestern Medical Center at Dallas, Dallas, TX, United States; ^3^Department of Neurology, University of Texas Southwestern Medical Center at Dallas, Dallas, TX, United States; ^4^Division of Infectious Disease, Department of Internal Medicine, University of Texas Southwestern Medical Center at Dallas, Dallas, TX, United States

**Keywords:** testicular cancer, cerebellar ataxia, limbic encephalitis, burned out tumor, germ cell tumor

## Abstract

A 44-year-old man presented with cerebellar ataxia and limbic encephalitis and was ultimately diagnosed with metastatic germ cell neoplasm resulting from a “burned out” primary testicular tumor. The patient had progressive ataxia, leading to a thorough investigation for infectious, autoimmune, metabolic, and malignant causes of acquired cerebellar ataxia that revealed no significant findings. Testicular sonography demonstrated a possible right testicular lesion that was not confirmed on radical inguinal orchiectomy. F18-FDG positron emission tomography/computerized tomography scan revealed a solitary retroperitoneal lesion, concerning for metastatic disease but not amenable to percutaneous biopsy. A robotic retroperitoneal lymph node dissection was performed and pathology revealed a CD117-positive metastatic seminoma leading to appropriate germ cell tumor-directed chemotherapy. After completing chemotherapy and during 1 year of follow-up, there has been a gradual improvement of the patient’s neurological manifestations.

## Introduction

Progressive cerebellar ataxia can be a component of paraneoplastic syndrome, therefore investigation for occult malignancy is warranted in singleton cases presenting with ataxia without known cancer. Malignancies commonly associated with paraneoplastic cerebellar degeneration include gynecological tumors, small cell lung cancers, and Hodgkin’s lymphoma, and rarely germ cell tumors in men (1).

We describe a case of a patient with metastatic germ cell tumor manifested initially as cerebellar ataxia with no primary testicular tumor found. This case underscores the potential pitfalls of occult malignancy workup in the setting of paraneoplastic neurological manifestations and emphasizes the methodological and multidisciplinary team effort needed in such cases.

## Case Presentation

**Figure 1:**
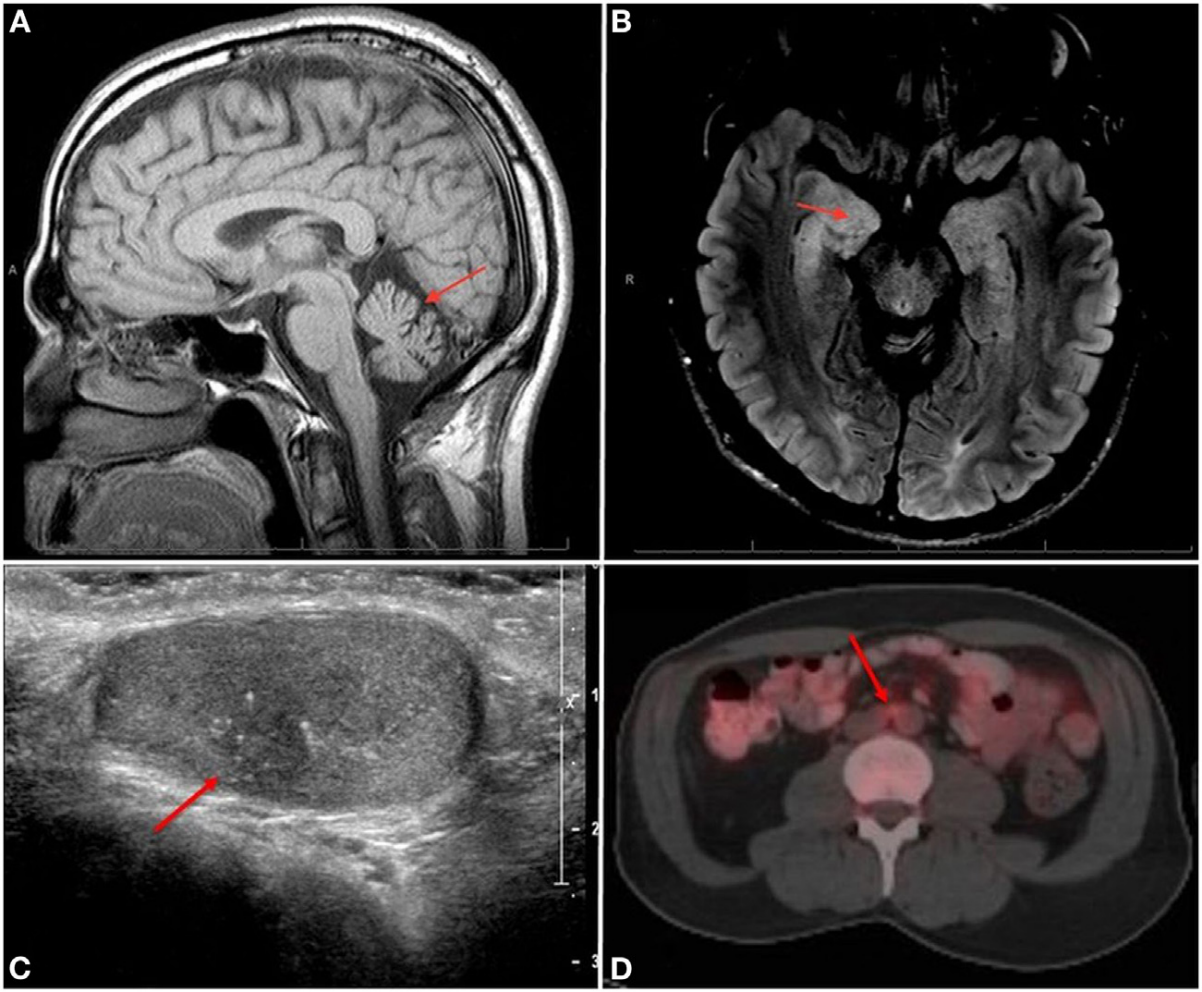
**(A)** Sagittal T1 image shows vermian atrophy (arrow) and **(B)** axial FLAIR image shows right hippocampal hyperintensity (arrow). **(C)** Right testicular US showing a 9 mm × 7 mm hypoechoic lesion (arrow) with microcalcifications. **(D)** F18-FDG positron emission tomography/computerized tomography showing an FDG-avid 1.4-cm × 0.9-cm lymph node in the periaortic chain (arrow).

In November 2016, a 44-year-old man presented to the neurology clinic with progressive gait and balance deterioration, slurred speech, and incoordination. The patient first noticed symptoms in the summer of 2015: he reported stumbling, feeling of balance, and shuffling when walking. A few months later, he noticed he was mumbling when speaking and had to repeat himself often in order to be understood. By 2016, all his symptoms had progressed. He started falling and noticed a left hand tremor. Family and personal history were unremarkable. The patient reported moderately alcohol consumption of approximately 8 drinks/day on weekends for about 20 years. Although he completely ceased alcohol consumption a year before presenting to the clinic, shortly after noticing the gait change, motor symptoms continued to progress. Initial neurological examination in our clinic revealed a wide-based gait and inability to perform tandem gait or stance (Video S1 in Supplementary Material). Saccadic dysmetria, and downbeat nystagmus was noted during upgaze but the remainder of cranial nerves were normal. Speech was dysarthric. He was hyporeflexic throughout but strength, tone, and sensation were normal. Remainder of the physical exam and mental status were without deficits. Initial brain magnetic resonance imaging scan (MRI) showed cerebellar vermian atrophy (Figure 1A).

Due to the progression of ataxia despite alcohol cessation, investigation for other acquired causes of cerebellar ataxia was initiated which included comprehensive metabolic tests, infectious disease serologies (*Coccidioides*, cryptococcal, HIV, *Histoplasma*, Lyme disease, *Tropheryma whipplei*, VDRL), paraneoplastic panel, toxicology (including alcohol levels), and vitamin (including thiamin levels—151 nmol\L) level screens. All tests were normal except for slightly elevated vitamin B6 and B12 levels. A whole body computerized tomography (CT) scan was obtained searching for potential neoplasm causing paraneoplastic ataxia. Neither chest nor abdominopelvic CT showed suspicious neoplastic lesions; however, the presence of pulmonary nodules of up to 0.3 cm prompted a Quantiferon TB gold test which was positive. Latent tuberculosis treatment was initiated with isoniazid.

Lumbar puncture revealed three nucleated cells, normal protein and normal glucose, but IgG index of 1.19 MML (normal <0.85 MML), IgG synthesis rate of 17.84 mg (normal ≤12 mg/24 h), 8 IgG oligoclonal bands (normal <4 bands) suggesting abnormal intrathecal IgG synthesis from an infectious, inflammatory, or immune-mediated process. Comprehensive cerebrospinal fluid (CSF) and serum infectious, paraneoplastic and autoimmune encephalopathy panels (NMDA receptor Ab, Neuronal VGK Ab, Glutamic acid decarb 65 Ab, GAbA B receptor Ab, AMPA receptor Ab, ANNA 1-3 Ab, Antiglial neuronal nuclear Ab, Purkinje type 1-2\TR Ab, Amphiphysin Ab, N\P\Q Type Ca channel Ab, ACHR binding Ab, ACHR ganglionic Neuronal Ab, Camp5 titer, Striated muscle Ab), all results were normal including germ cell tumor-related anti-Ma2 and small cell lung carcinoma-associated antineuronal-nuclear 1 antibodies. Serum tumor markers for testicular cancer (alpha fetoprotein, lactate dehydrogenase, and human chorionic gonadotropin) were normal. A decision to infuse intravenous (IV) immunoglobulin for a potential remote immune-mediated neurological disorder was made. IV steroids were avoided due to latent tuberculosis. Scrotal ultrasound to evaluate for occult testicular malignancy and body positron emission tomography (PET) scan to look for occult visceral malignancy were scheduled.

While awaiting outstanding diagnostic studies, 2 months after the initial consultation, the patient was admitted to hospital due to rapid alteration in mental status over 1–2 days and generalized tonic–clonic seizures. An electroencephalogram showed right temporal seizures. He was stabilized and treated effectively with antiepileptic drugs until seizures resolved. Brain MRI showed non-enhancing hyperintensity and swelling of right amygdala as well as hyperintensity in the periaqueductal gray area (Figure 1B). Although these findings are nonspecific and can be seen in disorders such as Wernicke’s encephalopathy, given the rapid occurrence of global encephalopathy, seizures, and abnormal CSF findings, the primary concern was limbic encephalitis from a potential paraneoplastic syndrome. Therefore, a 5-day course of plasma apheresis was initiated at the end of which his neurological course remained stable.

**Figure 2:**
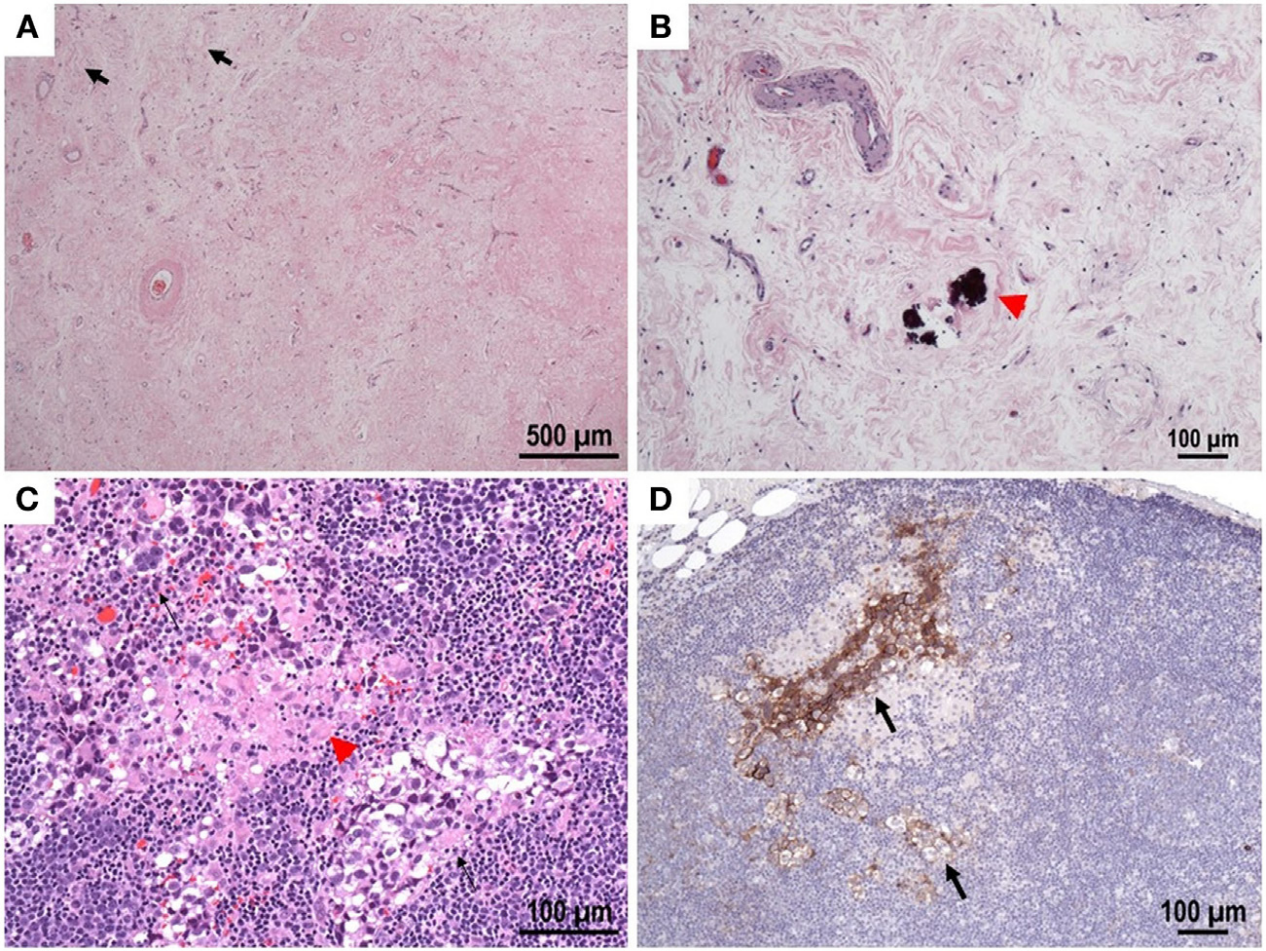
**(A,B)** Testis, hematoxylin and eosin stain: scarred area with hyalinized tubular Ghosts (lack arrow), increased vascularity and coarse calcifications (red arrow) within tubular profiles. No viable tumor was identified. **(C)** Lymph node, hematoxylin and eosin stain: small foci of metastatic GCT with seminomatous component (black arrows) with associated granulomas (red arrow head). **(D)** Immunohistochemical reactivity in tumor cells for CD117 support the diagnosis. CD30 (not shown) is negative.

A testicular ultrasound done in hospital showed a worrisome mass measuring 9 mm × 7 mm in the right testicle (Figure 1C). A right inguinal radical orchiectomy was performed, with pathological findings showing a 1.3 cm scar tissue with fibrosis and microcalcifications without sign of viable tumor or intratubular germ cell neoplasia *in situ* (Figure 2A). Although these findings were deemed nonspecific, a regressed germ cell tumor remained a concern.

Although a previous CT of the body was normal, given the concern for occult malignancy, an F18-FDG PET/CT scan was pursued which revealed a 1.4-cm × 0.9-cm solitary hypermetabolic lymph node in the interaortocaval distribution, worrisome for metastatic disease but not amenable to percutaneous biopsy (Figure 1D). A robotic retroperitoneal lymph node dissection was performed and pathology revealed a CD117 positive metastatic seminoma—stage IIA (Figures 2B–D) most probably secondary to a primary testicular “burned out” tumor. Four cycles of Etoposide and Cisplatin were initiated and completed. After completion of chemotherapy, the patient demonstrated no evidence of malignant disease. To date (Video S1 in Supplementary Material), with 1 year of follow-up after completion of therapy ataxia persisted although with some gradual improvement, the patient is currently able to walk with a cane although still suffers from occasional falls, speech and fine motor skills have slightly improved as well, no new seizures were reported.

## Discussion

Rapidly progressive cerebellar ataxia and limbic encephalitis might be autoimmune mediated or due to a paraneoplastic syndrome. Various malignancies have been associated with paraneoplastic neurological syndromes and must be considered in their differential diagnosis. Encephalitis might predate the actual diagnosis of malignancy by months, and delayed diagnosis may worsen both neurologic and oncologic prognosis. A thorough workup is warranted in each case (2), including periodically repeating diagnostic tests even when initial tests are normal (3). Onconeural antibodies such as Ma2 (commonly associated with testicular germ cell tumors) may point toward the possible malignant etiology (4) but their absence does not exclude it. Although not routinely recommended for germ cell tumor screening (2), a timely FDG PET/CT scan, which has a specificity and sensitivity of 67 and 80% for detecting malignancy, might be a useful initial screening method (5) for metastatic germ cell tumor causing cerebellar ataxia and limbic encephalitis in a previously healthy man.

Most retroperitoneal germ cell tumors represent metastatic spread from primary testicular lesions; however, primary retroperitoneal germ cell tumors have been reported. Burned-out testicular tumors are rare and manifest as completely regressed neoplasm on an orchiectomy specimen with metastatic spread. The cause of primary lesion regression is not clear and might be due to ischemia or an immune process (6). Paraneoplastic syndromes have been described in association with germ cell tumors; however, such a finding in the presence of a “burned out tumor” is an extremely rare occurrence with only two case reports to date to the best of our knowledge (7, 8). In one of them, diagnosis was only achieved after 4 years despite an extensive workup.

## Concluding Remarks

Paraneoplastic syndrome secondary to metastatic germ cell neoplasm from a “burned-out” testicular tumor is a rare occurrence (7, 8), but this case underscores the importance of a high clinical index of suspicion for testicular malignancy in men with symptoms of progressive cerebellar ataxia and limbic encephalitis.

## Ethics Statement

Written and informed consent was obtained from the participant for publication of this case report.

## Author Contributions

Drafting of the manuscript and literature search: YF, PKh, and AB. Figure acquisition: YF, PKa, PKh, and AB. Critical revisions: YF, PKa, RC, FL, PKh, and AB. Supervision: PKh and AB.

## Conflict of Interest Statement

The authors declare that the research was conducted in the absence of any commercial or financial relationships that could be construed as a potential conflict of interest.
